# Surgical Club of South West England

**Published:** 1984-10

**Authors:** 


### Surgical Club of South West England

### Spring Meeting held in Jersey on 4th and 5th May 1984

The first three papers were given by our Jersey hosts; not being strictly scientific papers abstracts have not been obtained. Nevertheless they were of considerable interest and the following has been compiled from notes taken at the time.

Mr. Roger Mitchell opened the proceedings with a talk about 'Jersey's Past and Present History'. The Channel Isles are the only part of the Dukedom of Normandy remaining to the heirs of William the Conqueror. Jersey French is the language of William the Conqueror, and is unintelligible to the French. The Channel Isles were formerly in the diocese of Coutance but are now in the diocese of Winchester. Jersey has its own parliament— the States of Jersey and makes its own laws. Jersey was Royalist in the Civil War and sheltered Charles II— 'This island has nothing to do with Parliament, only the King.' There is a police force on the island but they cannot make arrests, this can only be done by the Vingtonnierres. The only invasion attempted by the French was in 1781 when they were repulsed by the garrison commanded by Major Pearson who was himself killed in the encounter. In 1793 a force of 20,000 was assembled at St. Malo but was never actually launched. In 1940 the Germans invaded the islands but were unopposed. In 1944–45 the islands were isolated and Germans and islanders alike came near to starvation.

The population of the islands is 80,000 and they entertain a million visitors every year. They are not in the NHS and a Public Health committee runs their health service. General practice is private and there is one practitioner per thousand of the population. They charge £25 for a visit at weekends and average £25,000 p.a. after tax. Income tax is 20% and there is no capital transfer tax. In return for looking after U.K. visitors the Jersey Health service has special facilities in the U.K., e.g. Radiotherapy. Blood transfusion of rare groups is a problem and ruptured aneurysms are helicoptered to Bournemouth.

Mr. K. R. De demonstrated a patient who had been kept alive for 3½ years in good health on 'Total Parenteral Nutrition'. A girl of 19 had suffered a volvulus of bowel due to congenital incomplete rotation of the gut resulting in gangrene. It had been necessary to resect the entire small bowel and the ascending colon. She is fed through a subclavian catheter with pump rather than gravity feed. She receives Aminoplex, Glucoplex 1000 and 1600 and Intralipid with vitamins and zinc sulphate. Due to zinc deficiency her hair turned blonde. She takes only clear fluid orally; she once tried a full dinner with dire results. The annual cost of her nutrient bags is £20,000. The feeding takes 10 hours a day. At one time she gained weight, now she remains steady on 1800 cal per day. She works in the hospital as a cashier clerk.

Dr. D. Spencer gave a talk entitled 'A Little Pathological "Fancy That"'. He described 3 cases that had come to autopsy. The first lady who had had an intestinal bypass 5 years prior to becoming pregnant and had died 3 months post partum of a cardiomyopathy. The second was a man accustomed to drinking 2½ bottles of brandy a day. He suddenly stopped drinking, went into D.T.s and died in 24 hours. Autopsy revealed two occipital fractures 4½ inches apart. The cause of these fractures was only settled when the taps in his bathroom were measured and found to be 4½ inches apart. The last was a patient admitted after stating that he had taken an overdose of Valium. In spite of all efforts he became unconscious and died. At autopsy his blood was liked liquid chocolate and analysis revealed methaemoglobinaemia. He was a retired gardener and had taken a dose of potassium chlorate weed killer. He must have given the false information about taking Valium to frustrate attempts to save him.

M. G. W.

### USE OF THE ANGELCHIK PROSTHESIS TO PREVENT OESOPHAGEAL REFLUX

B. C. Clendinnen, Jersey General Hospital

The Angelchik anti-reflux prosthesis was demonstrated with a brief historical introduction. The causes of reflux were reviewed with diagrams. Attention was drawn to the different mechanical factors obtaining when intragastric pressure increases due to increasing intragastric volume in contrast to increases of extragastric pressure.

The prosthesis is believed to prevent reflux by interrupting the pull of the stomach wall to distract the cardia. This happens in accordance with Laplace's law as increasing intraluminal gastric radius increases gastric wall tension. Experimental support for this hypothesis was acknowledged (Samuelson et al., *Ann.Surg.* 1983, **197**, 254).

It is stressed that the prosthesis can only prevent reflux where normal anatomical relations at the hiatus can be restored.

The author has used the prosthesis with increasing confidence but did not dwell on the results as this paper introduces a controlled trial of this procedure compared with Nissen fundoplication (next paper).

Complications of the procedure and operative details were discussed.

This simple operation could become the procedure of first choice to control reflux where the cardia can be brought below the diaphragm. If unsuccessful it is probable that the prosthesis may be removed without difficulty and without having caused harm.

### RANDOMISED PROSPECTIVE TRIAL OF THE ANGELCHIK ANTI-REFLUX PROSTHESIS

**Michael W. L. Gear, E. Walford Gillison and Brian L. Dowling, Gloucestershire Royal Hospital, Kidderminster General Hospital and Northampton General Hospital**

Fifty-two patients with reflux oesophagitis resistant to medical treatment were randomised at operation to receive either the new Angelchik prosthesis or a fundoplication. All patients were assessed post-operatively by a physician unaware of the nature of the operation. Forty-two patients have been followed up for 1 to 2 years; 10 patients for 3 to 9 months.

Of the Angelchik patients 96% had satisfactory or excellent results compared with 81% with a fundoplication. There were no failures of reflux control with the Angelchik prosthesis whereas six patients (23%) of the fundoplication group have persisting reflux. Operating times for insertion of the prosthesis averaged a little over half that recorded for fundoplication. Complication rates were similar.

The results of the trial encourage the use of the prosthesis in patients with gastro-oesophageal reflux, where medical treatment has failed.

The prosthesis should not be used if the gut is opened during operation either inadvertently or deliberately, as in making a suture line or anastomosis, because of the risk of sepsis.

### OESTROGEN RECEPTOR ASSAY—THE WAY FORWARD?

**A. C. Akehurst, Musgrove Park Hospital, Taunton**

The surgical dilemma in treatment of carcinoma of the breast was emphasised, and the current pressure to join clinical trials of breast carcinoma discussed. It was pointed out that a large number of cases presenting to the clinician do not fit into any of the current trials, making it difficult to collect significant numbers and perhaps affecting statistical results.

Many trials do not include oestrogen receptor assays, and it was felt that this omission might well invalidate the conclusions of such trials. Oestrogen receptors are now being carried out routinely in the Taunton and Somerset Hospital, and have proved satisfactory and reasonably cheap at £10 per test. In the past $2\frac{1}{2}$ years about 63 assays have been reported. In this limited number of cases the test has proved a useful prognostic indicator, and appears to support the conclusions of Tim Cooke's paper of 1980.

It was suggested that efforts should be made to set up a Breast Tumour Registry in the South West Region to include all cases of proven breast carcinoma, with computerised records for future analysis.

### TRIALS OF ANTIBIOTIC PROPHYLAXIS OF WOUND INFECTION

**D. J. Leaper, M. J. Cooper and W. E. G. Thomas, University Department of Surgery, Bristol Royal Infirmary**

Progression in research for perfection in surgical wound closure without the complication of infection has almost attained one of Lord Lister's life's objectives. The most important single determinant of postoperative wound infection is endogenous bacterial contamination of the wound when the gastrointestinal tract is opened. At the Bristol Royal Infirmary patients undergoing this surgery have been entered into prospective random controlled clinical trials.

In upper gastrointestinal surgery cefotetan was found to be an effective safe antibiotic in eliminating wound infections and was significantly better than cefazolin in reducing postoperative chest and urinary infections ($P<0.05$). Cefotetan is now being compared with the acylureide penicillin mezlocillin. Ceftriaxone was equally effective in a separate open trial and is now being assessed in lower gastrointestinal surgery. Moxalactam was more effective in colorectal surgery than a dual combination of metronidazole and cefuroxime given as three doses postoperatively. Wound infections occurred in 10–15% of patients overall and sepsis was minor in nature.

The new cephalosporins are increasing in their effectiveness against a wider range of organisms. They are a safe group of antibiotics which present an increasing selection with unpronouncable names. We have found them to be entirely suited to wound sepsis prophylaxis during gastrointestinal surgery. It would be unwise to dismiss them, as their generations and number continue to increase, as the cephalo-borins!

### CRYO SURGERY IN ADVANCED MALIGNANT DISEASE

**H. Reece-Smith and K. Lloyd Williams, Royal United Hospital, Bath**

Cryo surgery has been used extensively for the treatment of haemorrhoids and superficial skin lesions. In this study we have used freezing for the palliative treatment of advanced malignant disease, using the Spembly liquid nitrogen apparatus.

Fifteen locally recurrent breast tumours that had failed to respond to radiotherapy, hormone manipulation and chemotherapy have been treated. Good symptomatic results were obtained in eight. Seven patients either failed to respond or were left with a discharging cavity that failed to heal.

Thirteen patients with carcinoma of the rectum were treated initially with cryo therapy because of their poor general condition. Seven had relief of all symptoms, four were considered treatment failures. The remaining two were treated by abdomino-perineal resection after cryo surgery; one died 3 years later from pneumonia, the other was a postoperative death.

Two patients with recurrent tumour after anterior resection were rendered asymptomatic with cryo surgery but perineal recurrence after A-P resection did not respond so well with only two out of five patients gaining significant symptomatic benefit.

Skin secondaries from a variety of primary tumours did not respond well to cryo therapy but a number of less common tumours and three cases of malignant melanoma have been treated with a reasonable response rate.

### THE ENDOSCOPIC TREATMENT OF UPPER URINARY TRACT STONES

**J. C. Gingell and J. P. Penry, Southmead Hospital, Bristol**

Urology is now on the threshold of major changes in the surgical management of stone disease, almost certainly to have as big an impact on urological practice as the transition from open prostatectomy to transurethral resectiom.

It is with some embarrassment to the surgeon and considerable discomfort to the patient that stones of less than 2 cm in diameter in the renal pelvis have to date required such a large and painful loin incision for their removal. An alternative technique of percutaneous removal of such stones has been developed. Under screening the patient's kidney is needled by the radiologist and a guide wire is inserted into the renal pelvis under IV sedation. Specially designed graduated dilators are passed down the wire until a neoplex catheter of between 24 and 27 Charriere can be inserted into the pelvis. Several days later when the tract has matured and the urine is clear, the patient is returned to the X-ray Department, and under intubated anaesthesia the catheter is removed and the endoscope passed into the kidney alongside the guide wire. The stone is readily located, grasped in suitable forceps or basket and extracted. One or two skin satures are inserted, and the patient is usually discharged the next day. Stones too large to be pulled down the tract can be fragmented by ultrasonic lithotripsy using a contact probe passed down the endoscope or by electro-hydraulic shock waves similarly delivered directly to the stone by a probe. The resulting fragments are irrigated free or extracted.

Urologists have always been justifiably reluctant to use the Dormia basket to remove stones impacted in the ureter above the pelvic brim. The development of a rigid uretero-renoscope now allows such stones to be extracted safely under direct vision. Our initial satisfactory experience of these techniques for endoscopic removal of upper tract stones at Southmead was reported.

### VIDEOPROSTATECTOMY—THE STATE OF THE ART 1984

**P. J. O'Boyle, G. N. Lumb and G. V. Appleton, Department of Urology, Musgrove Park Hospital, Taunton**

The value to the patient with outflow tract obstruction of a transurethral operation conducted by a trained endoscopic surgeon is beyond dispute. The position for the surgeon is less clear. Transurethral surgery requires considerable concentration and may be, physically, extremely demanding. This is partly due to the unnatural postures which the surgeon is required to adopt in his quest for the complete prostatectomy.

Recent advances in optical technology have now made feasible the prospect of conducting transurethral surgery using a micro-video camera to project the endoscopic image on a video monitor. This means that the surgeon can now operate in a position of comfort with the hands below the shoulder level—'The armchair prostatectomy'. The details of technique and the advantages to the surgeon, to the anaesthetist and indeed to all theatre personnel, were discussed. The degree of involvement of the theatre staff makes the acquisition of the technique most rewarding.

### ABDOMINAL WALL TENDERNESS TEST

**S. St. C. Carter and W. H. F. Thomson,**
**Gloucestershire Royal Hospital**

From 1975 to 1983 a record was kept of patients referred with atypical abdominal pain in whom the abdominal wall tenderness test was carried out. The test is positive when palpation at a certain spot is more tender with the abdomen tensed than relaxed. A study of the notes of 73 such cases, together with the General Practitioners' records in 50 of them, forms the basis of this report.

The great majority were females between 20 and 50 years old. They were characterised by a past history of multiple hospital admissions, a story of low back pain (40%), depression or anxiety requiring treatment (20%), appendicectomy (32%) and hysterectomy (29%).

Seventy-two per cent had had X-ray investigation, multiple and often repetitive in 40%, with normal results in the great majority.

The records showed continuing complaint of the pain in 68%, often despite laparotomy, scar exploration, hysterectomy and D & C. In only two patients did events show the pain to have a certain organic origin: in one spinal metastases from uterine carcinoma (previously removed), in the other, chronic pyelonephritis. The ESR was raised in the first, the MSU abnormal in the second.

We conclude that when the test is positive in someone with atypical abdominal pain, it is usually indicative of intraperitoneal innocence. Experience in its use will save the cost, inconvenience, and occasional danger of sophisticated investigation, and prevent some unnecessary laparotomies.

